# Stability Evaluation and Degradation Studies of DAC^®^ Hyaluronic-Polylactide Based Hydrogel by DOSY NMR Spectroscopy

**DOI:** 10.3390/biom10111478

**Published:** 2020-10-24

**Authors:** Tatiana Guzzo, Fabio Barile, Cecilia Marras, Davide Bellini, Walter Mandaliti, Ridvan Nepravishta, Maurizio Paci, Alessandra Topai

**Affiliations:** 1 Colosseum Combinatorial Chemistry Centre for Technology S.r.l (C4T), Via della Ricerca Scientifica snc, 00133 Rome, Italy; tatiana.guzzo@c4t.it (T.G.); fabaril@gmail.com (F.B.); cm.ceciliamarras@gmail.com (C.M.); 2 Novagenit Srl, 38017 Mezzolombardo (TN), Italy; d.bellini@novagenit.com; 3 Department of Chemical Science and Technology, University of Rome, Tor Vergata, 00133 Rome, Italy; w.mandaliti@alice.it (W.M.); nepravishta@gmail.com (R.N.); paci@uniroma2.it (M.P.)

**Keywords:** DAC^®^ HA-PLA copolymers, biopolymer degradation, polymer stability, DOSY NMR

## Abstract

The stability and the degradation of polymers in physiological conditions are very important issues in biomedical applications. The copolymer of hyaluronic acid and poly-D,L-lactic acid (made available in a product called DAC^®^) produces a hydrogel which retains the hydrophobic character of the poly-D,L-lactide sidechains and the hydrophilic character of a hyaluronic acid backbone. This hydrogel is a suitable device for the coating of orthopedic implants with structured surfaces. In fact, this gel creates a temporary barrier to bacterial adhesion by inhibiting colonization, thus preventing the formation of the biofilm and the onset of an infection. Reabsorbed in about 72 h after the implant, this hydrogel does not hinder bone growth processes. In the need to assess stability and degradation of both the hyaluronan backbone and of the polylactic chains along time and temperature, we identified NMR spectroscopy as a privileged technique for the characterization of the released species, and we applied diffusion-ordered NMR spectroscopy (DOSY-NMR) for the investigation of molecular weight dispersion. Our diffusion studies of DAC^®^ in physiological conditions provided a full understanding of the product degradation by overcoming the limitations observed in applying classical chromatography approaches by gel permeation UV.

## 1. Introduction

Important new advances have been reported about new materials and biomaterials [1]. In the field of biomaterials, polymers and copolymers have found large application in modern medicine [2]. Biodegradability of polymeric biomaterials constituted a significant advantage, being that these materials are able to be broken down and removed when they have exerted their function [2]. Clinically, there are a wide range of applications of degradable polymers, such as surgical sutures and implants. Desired physical, chemical, biological, biomechanical, and degradation properties can be selected and tuned in order to fit all the requirements of the functional demand.

Fortunately, novel materials are developed constantly to meet new challenges providing a growing number of natural and synthetic degradable polymers, investigated for biomedical applications. Particularly, degradable polymers are of interest since these biomaterials are able to be molecularly broken down and eliminated or resorbed without physical removal or surgical revision [3].

In the design of biodegradable biomaterials, great importance should be given to their properties [3]. In fact, these materials should not produce a sustained inflammatory response and have a degradation time coinciding with their function. Moreover, they should have appropriate mechanical properties for the scope, produce non-toxic degradation products that can be readily resorbed or excreted, and include appropriate permeability and processability for the designed application [3].

Orthopedic and traumatology (O&T) are up to 38% of the worldwide leading markets of implanted biomaterials [4], involving each year millions of new patients with an increasing trend. Infections related to implanted medical devices depend on the bacterial capability to establish highly structured multilayered biofilms on artificial surfaces and represent the most devastating complication in O&T, with millions of cases. In many cases, a slow release of antibiotic [5] can help the success of the intervention.

**Figure 1:**
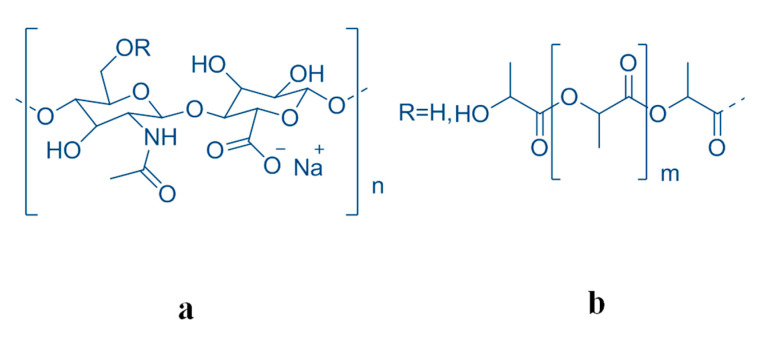
The molecular moieties (**a**) hyaluronic acid (HA) and (**b**) polylactic acid (PLA) forming the copolymer HA-PLA (b/a + b = 13% *w*/*w*).

The aim of the present work is to assess the stability and the molecular degradation of a disposable coating of the implanted biomaterial Defensive Antibacterial Coating (DAC). Produced in the form of a powder, this device must be hydrated with water for its preparations, and is injectable alone or in solution, which is associated with an antibiotic to obtain a formulation in a hydrogel. The specific indication is for the prevention of peri-implant infection. This device, based on a novel resorbable hydrogel, would act as a resorbable barrier delivering local antibiofilm and antibacterial compounds. The active drugs will be mixed at the same time as the application of the hydrogel during surgery, allowing the correct choice for any given patient, reducing costs and improving storage life and versatility of use. This hydrogel is a derivative of hyaluronic acid (HA) which is present in large quantity in synovial fluid and vitreous humor, which contributes to these tissues’ viscoelastic properties and which plays an important structural role in articular cartilage. HA is a natural linear polymer with disaccharide repeating units, namely, [(1→4)-beta-d-GlcpA-(1→3)-beta-d-GlcpNAc]. Natively, HA has been extracted from a variety of animal tissues in the past. Due to the limited sources and a risk of viral infection, the extraction technique has been replaced by microbial fermentation with a high purification efficiency, low production cost, and low risk rate of cross-species viral infection [6,7,8,9]. Owing to its relevantly unique physico-chemical properties and its important biological activities as a drug carrier [10,11,12], HA has been used in a wide variety of applications, such as food, biomedicine, biomaterials, and cosmetics. The gels obtainable by using cross-linked HA chains by chemical derivatization led to several chemical modifications. The cross-linking has been proposed to achieve chemical and mechanical HA robustness [13,14,15]. In particular, the conjugation with poly-D,L-lactic acid (PLA) [4,16,17,18,19] (Figure 1) gives an ideal product for the predesigned pharmacological use.

In fact, this HA derivative is important for two main requisites to be reached. First, the stability of the hyaluronic acid chain against depolymerization, and secondly, the stability of the chemical junction between HA polymer and PLA moiety to confer to the polymer the desired physico-chemical characteristics. The sterilization procedure by ionizing through gamma ray irradiation induces a fragmentation of HA, as has been well reported [20]. The samples for the stability study have been synthesized by a new method property of Novagenit. The product has been named HA-PLA Novagenit DAC^®^ and obtained chemically in a sterile condition with an esterification of HA of about 13% (*w*/*w*) at the origin. The stability of the conjugate with sterile polylactic acid has been studied directly at the surface of titanium prosthesis as well as in the bulk region. For these samples, the physico-chemical characteristics, the stability of the copolymer, and its molecular weight dispersion have been studied by NMR spectroscopy [21] over time, which included a temperature cycle. The earlier proposed application of the diffusion-ordered spectroscopy (DOSY) NMR revealed it was particularly successful in this kind of study in order to distinguish among different molar diffusivities due to the different hydrodynamic radii that are often correlated with the different molecular weights [22,23,24,25]. In this field, very important advances have been made by DOSY NMR in general [26,27] for the separation of drugs in mixtures [28,29]. It is important that in similar studies addressed to the measure of precise molar diffusivity, should be performed usually with a gradient strength quite higher than that used in this study. Thus, the reported results are to be considered as apparent values that, notwithstanding, allowed us to monitor the macromolecular degradation and the extent of the hydrolysis of the conjugation. The characteristic of linear polymers (far from the usual spherical shape of macromolecules) led us to consider these results valid [30]. A discussion of the obtained results is detailed in a paragraph in the discussion section.

This study appears particularly important because of the diverse biological activities of the hyaluronan fragments [31]. In fact, depending on the polymer length, i.e., small, medium, and large fragments, small and medium ones have pro-angiogenic and anti-apoptotic properties which stimulate the synthesis of heat-shock proteins (HSP) as potent immunostimulants. Immunosuppressive and anti-angiogenic functions are mainly exerted by large polymers [12].

As reference standards with high molecular homogeneity, certified HAs at various molecular weights (MWs) have been used. This approach was already applied in other cases to reveal the presence of hyaluronic acid in mixtures [30]. In our case, this spectroscopic approach revealed to be more precise and sensitive than the results obtained by gel permeation chromatography reported below [32,33].

## 2. Materials and Methods

### 2.1. Materials

HA sodium salt HySilk reference standard with MW 13 kDa and 280 kDa were furnished by Giusto Favarelli (Via Medardo Rosso, 8, 20159 Milano, Italy), and HA sodium salt 50 kDa was purchased from Sigma-Aldrich (Milano, Italy). PLA “Purasorb” was purchased from Corbion Biomaterials Purac Biochem (Gorinchem, The Netherlands). HA-PLA in sterile and non-sterile form was prepared by Novagenit (Mezzolombardo-TN, Italy).

### 2.2. High Molecular Weight HA Sodium Salt

Sodium hyaluronate “high MW” with a value about MW ≥ 500 kDa for pharmaceutical and medical use was purchased from HTL (7 rue Alfred Kastler-ZI de l’Aumaillerie, 35133 Javené, France).

### 2.3. Synthesis of DAC^®^

DAC^®^ copolymer was synthesized by esterification of HA with PLA according to the experimental protocol already registered by Novagenit [20]. In order to allow the grafting of PLA to HA, PLA was reacted with carbonyl di-imidazole to obtain the corresponding imidazole derivative (PLA-CI). Subsequently, the reaction between carboxyl-activated PLA and an organic soluble HA form (Hyaluronic acid tetrabutyl ammonium salt, HA-TBA) is carried out in N-methyl pyrrolidone (NMP), 48 h, 37 °C; finally, precipitation and ion exchange lead to the sodium salt form of HA-g-PLA. The final derivatization grade is PLA/(HA + PLA) = 13% *w*/*w*.

### 2.4. Stability Tests

Sample preparation and stability tests were performed according to UNI EN ISO 10993-9 [34], ISO 10993-13 [35] and ISO 13871:1995 [36] guidelines. The test was performed in triplicate, and was designed over 5 study times (from a few seconds to 15 days). For each study time, DAC^®^ hydrogel was uniformly distributed over the roughened surface of the titanium disk. The system was topped with a second titanium disk, obtaining a “sandwich”. Each double disk system was immersed in PBS solution at pH 7.2, each container was closed and placed at 37 °C, without stirring, to simulate the physiological condition of the medullary cavity. At each study time, for each sample, the double disk system was separated from its buffered solution, and disk samples and solutions were freeze-dried separately. For the initial study time, sudden disk removal, no sample is recovered from the buffer solution. For the final study time, 15 days, no sample is recovered from the interface of the two disks. A total of 24 samples are therefore recovered for the High Performance Liquid Chromatography (HPLC) and NMR study (Table 1).

Sterile titanium disks 8.0 cm diameter and 0.4 cm thick were purchase by Adler Ortho (Via dell’Innovazione, 9, 20032 Cormano, Italy). Titanium disks 6.5 cm diameter and 0.4 cm thick were purchased by Adler and sterilized by gamma irradiation.

### 2.5. Degradation Method

The degradation method of a single batch of the original sample HA-PLA DAC^®^ from Novagenit is reported in the proprietary patent.

Degradation was obtained by 1.0 mL of NaOH 0.2 M solution heated at 60 °C, then 30 mg of sample is added stirring at constant temperature for 30 min. The aqueous phase is extracted from dichloromethane to eliminate free PLA produced by hydrolysis.

### 2.6. Analytical Methods

The chromatographic experiments (HPLC-UV) were performed by Agilent instrument 1260 Series. 2 Waters UltraHydrogel columns in series (UH 500, 10 μm, 7.8 × 300 mm and UH250, 6 μm, 7.8 × 300 mm) were eluted with microfiltrated water milliQ PBS at pH 7.2, at 0.8 mL/min flow/rate. 20 μL injection was used. UV detector was set at 200 nm.

### 2.7. NMR Spectroscopy Measurements

^1^H NMR were performed on Bruker Avance 300 MHz and Bruker Avance 400 MHz instruments. ^1^H Diffusion ordered NMR (DOSY) experiments were run on Bruker Avance 700 MHz. Monodimensional NMR spectra were achieved by single-pulse NMR using the solvent suppression only in the case of samples in water solution by using a 90degree pulse and a repetition time as to allow the magnetization to relax completely. The spectra were registered in 8K data point and then transformed in 16K data point. Deuterated solvent for lock purpose was purchased from Sigma Aldrich (Milano, Italy): DMSO-d6, 99.9% D, or D_2_O 99.9 atom % D. To estimate possible solvent effects on the spectrum of the sample other samples have been prepared in DMSO-d6–D_2_O mixture (DMSO-d6:D_2_O = 9:1) or (DMSO-d6:D_2_O = 8:2) containing all 7.5 mg of sample.

The characteristic resonances of PLA and HA sodium salt were identified: for PLA 3H δ 1.4–1.6 CH3, 1H δ 5.0 CH and for HA sodium salt 3H δ 1.8 CH3(CO-NH); 2H δ 4.5–4.62.

In a diffusion ordered spectroscopy (DOSY) experiment, a series of NMR spectra is normally acquired in a spin or stimulated echo as a function of pulsed-field gradient amplitude, with the amplitude of each signal decaying at a rate determined by the diffusion coefficient. The ideal behavior for unrestricted diffusion is described by the Stejskal–Tanner equation [25].

The diffusion ordered spectroscopy (DOSY) experiments were performed by using the ledbpgppr2s pulse sequence of the Bruker library also in order to suppress the water signal at 4.7 ppm, when necessary. During the DOSY experiment 32 mono dimensional spectra were acquired with 64 scans in a linear increasing gradient varying from 5% to 95% with a pulsed gradient time δ of 70 ms and a diffusion time Δ of 2 ms. The spectra were then analyzed using the DOSY module implemented in Bruker software TOPSPIN 3.1 (Bruker Italy, Milano, Italy).

## 3. Results and Discussion

The samples were from the same batch of the original sample HA-PLA DAC^®^ from Novagenit obtained as reported in the proprietary patent. The samples have been sterilized as indicated in the preparation protocol. The degree of degradation was obtained as reported in Materials and Methods.

### 3.1. NMR Spectroscopy Study

**Figure 2:**
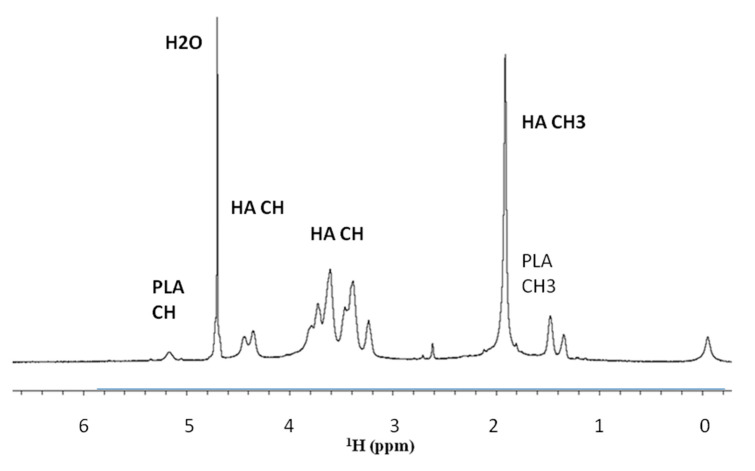
NMR spectrum for HA-PLA DAC^®^ sterilized HA sodium salt–PLA at a concentration of 7.5 mg/mL in D_2_O-PBS obtained as reported in Materials and Methods.

The NMR spectra of the HA sodium salt were obtained as reported in Materials and Methods. The observed chemical shifts were aligned with the literature data [8] and the integral of the signals (3H δ 2.08 CH3(CO-NH); 2H δ 4.5–4.62) showed the correct ratio of protons resonances. In total 16H (3 + 11 + 2). For PLA (3H δ 1.4–1.8 CH3;1H δ 5.2–5.4 CH, with a correct ratio between the resonance integrals 3H:1H). A typical spectrum of HA-PLA is reported in Figure 2.

The samples of HA-PLA DAC^®^ sterilized were preliminary studied in D_2_O as to reduce the large water resonance dissolving at a 7.5 mg/mL concentration. The NMR resonance assignment was achieved by literature data distinguishing between HA and HA-Na salt. A mixture of D_2_O-DMSO was also examined. In fact, in the NMR spectra, a marked dependence of the PLA resonances upon the different solvent has been observed. In the solvent mixtures DMSO-d6–D_2_O (DMSO-d6:D_2_O = 9:1) or (DMSO-d6:D_2_O = 8:2) an increase in intensity of the PLA resonances with respect to the HA-Na resonances has been observed at the same concentration, with an increase of the intensity of the HA resonances upon the increase of the D_2_O ratio (reported in Supplementary Materials, Figure S1).

During this study, the possibility of micellar aggregation in solvents was also considered. In fact, in the mixtures of water and DMSO at different ratios, it is easy to detect a probable formation of micelles. PLA is much more soluble in the non-aqueous system while HA strongly prefers the aqueous one. This effect has been already reported with copolymers like HA-PLA [17,33].

The stability of the presence of the conjugate between HA and PLA needed to be proved after sterilization. In fact, there was the possibility that the spectrum of Figure 2 would result as a simple additive overlap between the NMR spectrum of the HA and PLA. In our case, the DOSY experiment of HA-PLA after sterilization showed a unique diffusive front and, thus, confirmed the presence of a unique product (HA-PLA) chemically linked together. The observed diffusive front was 8.91 × 10^−11^ m^2^ s^−1^.

**Figure 3:**
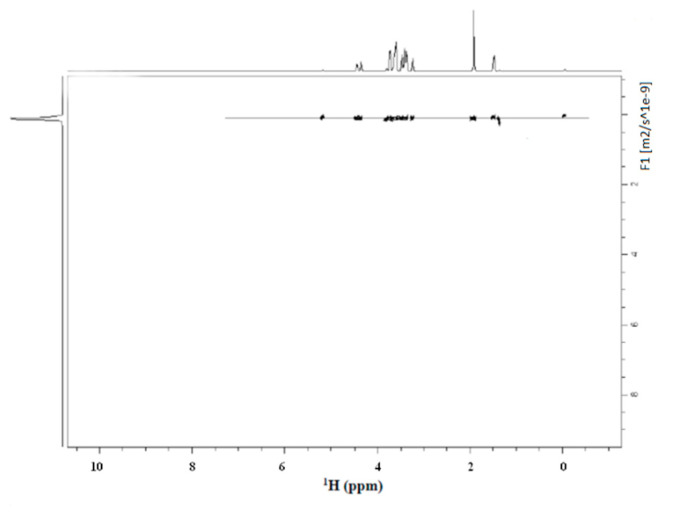
Diffusion ordered NMR (DOSY) experiment of the sample HA-PLA DAC^®^ in D_2_O-PBS after sterilization. The front of diffusion indicated a Diffusion coefficient D = 8.91 × 10^−11^ m^2^ s^−1^. A weak resonance is reported at 0 ppm probably due to the silyl moiety of DSS bound to the macromolecule.

Moreover, DOSY experiments reported in Figure 3 for sterilized HA-PLA DAC^®^ obtained as reported in Materials and Methods revealed that no differences are present in hydrodynamic volumes. This indicates there is only one population with the same diffusion coefficient and a unique molecular weight for the HA-PLA copolymer. Particularly the sharp shape of the peak on the left side of the DOSY representation indicates that nearly a unique species is present in the solution for the observed diffusive front. A weak diffusion is visible at 0 ppm probably due to a trace of the silyl moiety of DSS bound to the macromolecule, due to hydrophobicity of PLA moiety.

**Figure 4:**
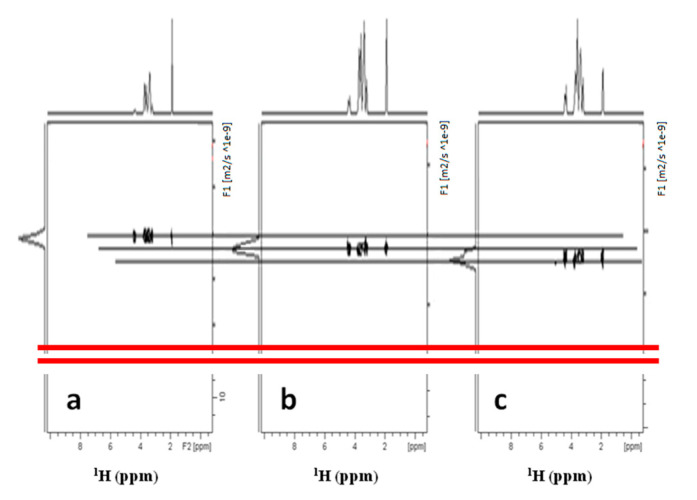
DOSY experiments of three different HA polymer standards in D2O with different MWs. (**a**) 13 kDa; (**b**) 50 kDa; (**c**) 208 kDa. To avoid confusion due to possible interferences, the experiments were run on single polymer samples. The diffusion coefficient obtained are used for the calibration curve in the log-log plot of the Figure 5.

**Figure 5:**
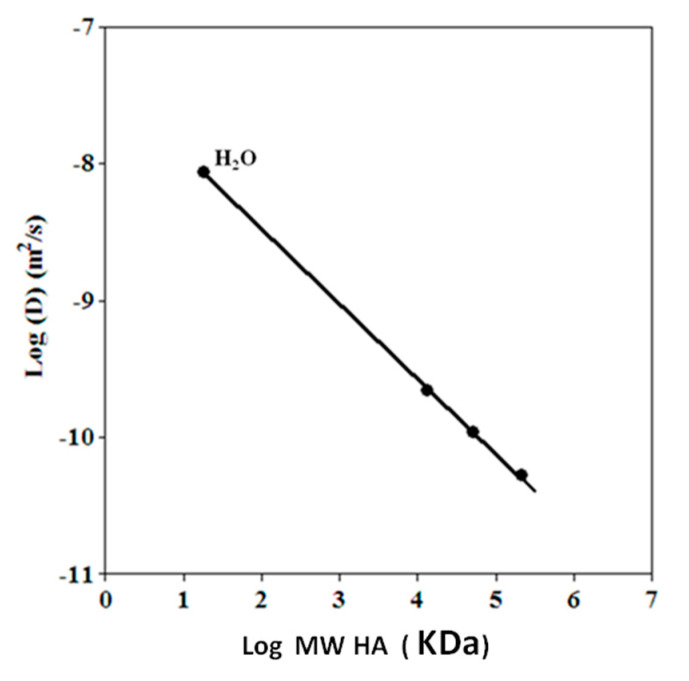
Calibration curve of the diffusion coefficient of fragments of HA with different MWs: 13 kDa; 50 kDa; 208 kDa. The diffusion of the trace of protonated water molecule (1% in D_2_O) is also included with a correlation of 0.999.

Thus following the recommendations [29] of using standards with sufficient molecular homogeneity to calibrate the MW, a series of DOSY experiments were conducted on separate solutions of HA fragments with different MWs (13 kDa, 50 kDa, 208 kDa), to check the linearity of hydrodynamic radii and the MWs in hyaluronans. The results are reported in Figure 4 with a final log-log plot in Figure 5 reporting the good correlation found. An investigation was also performed to ascertain if an interference of macromolecules of different sizes in the measure of diffusion could occur. These results are reported in more detail in Supplementary Materials and in a dedicated section of the Discussion.

The calibration curve of standards is reported in the following Figure 5, with a correlation coefficient of 0.999. The inspection of the results of the calibration curve indicated that the experiment is able to distinguish between fragments with MW lower than 13 kDa (from 5.0 to 13 kDa), in line with the expected range of MWs in the degradation experiments. Furthermore, the DOSY results included the resonance of protons of residual water, whose diffusion value displayed a good alignment with those of the fragments of HA observed. A more detailed discussion of the diffusion results is reported in the related paragraph in the Discussion section.

Moreover, a detailed study of the possible interferences between these types of polymers was carried out using a mix of the 13 kDa with the 50 kDa ones and of the 13 kDa with the 208 kDa standard samples. In addition, these results are reported in Supplementary Materials and in the related paragraph in the Discussion section.

### 3.2. Stability Studies of HA-PLA DAC^®^ Sterilized

**Figure 6:**
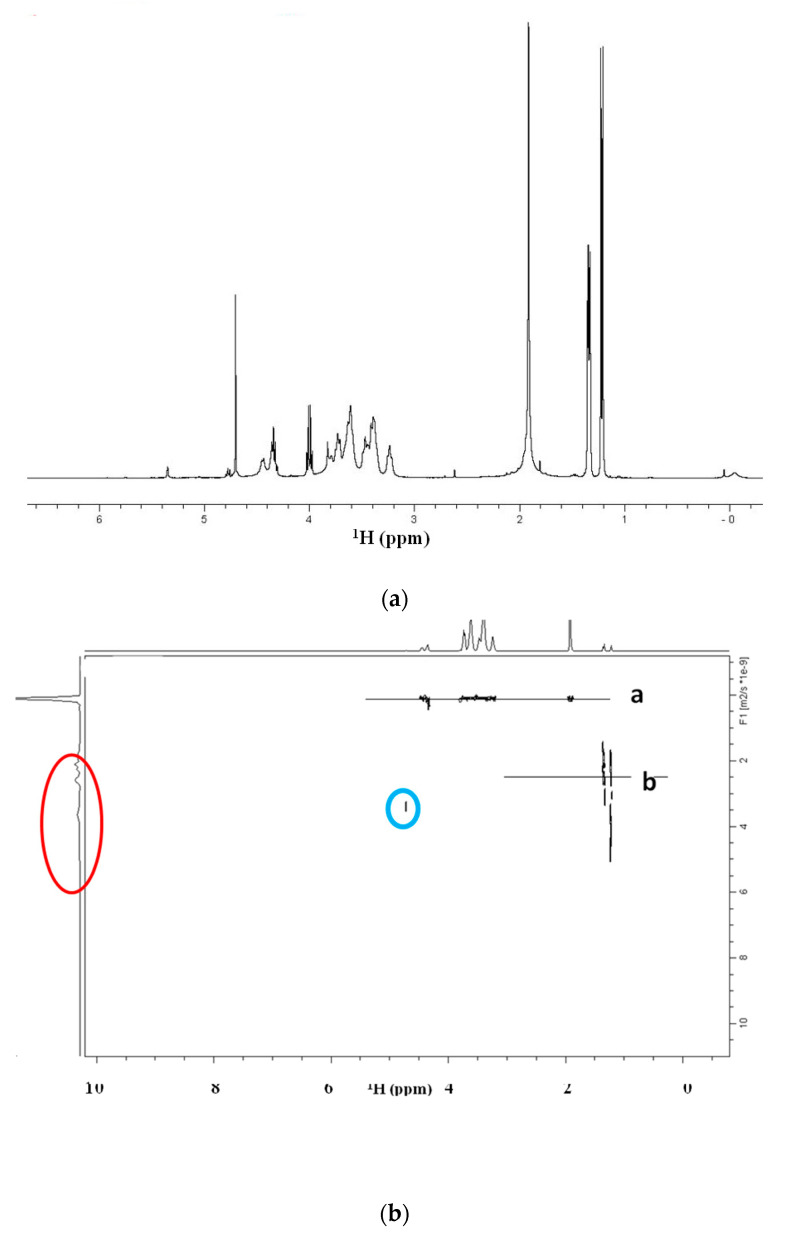

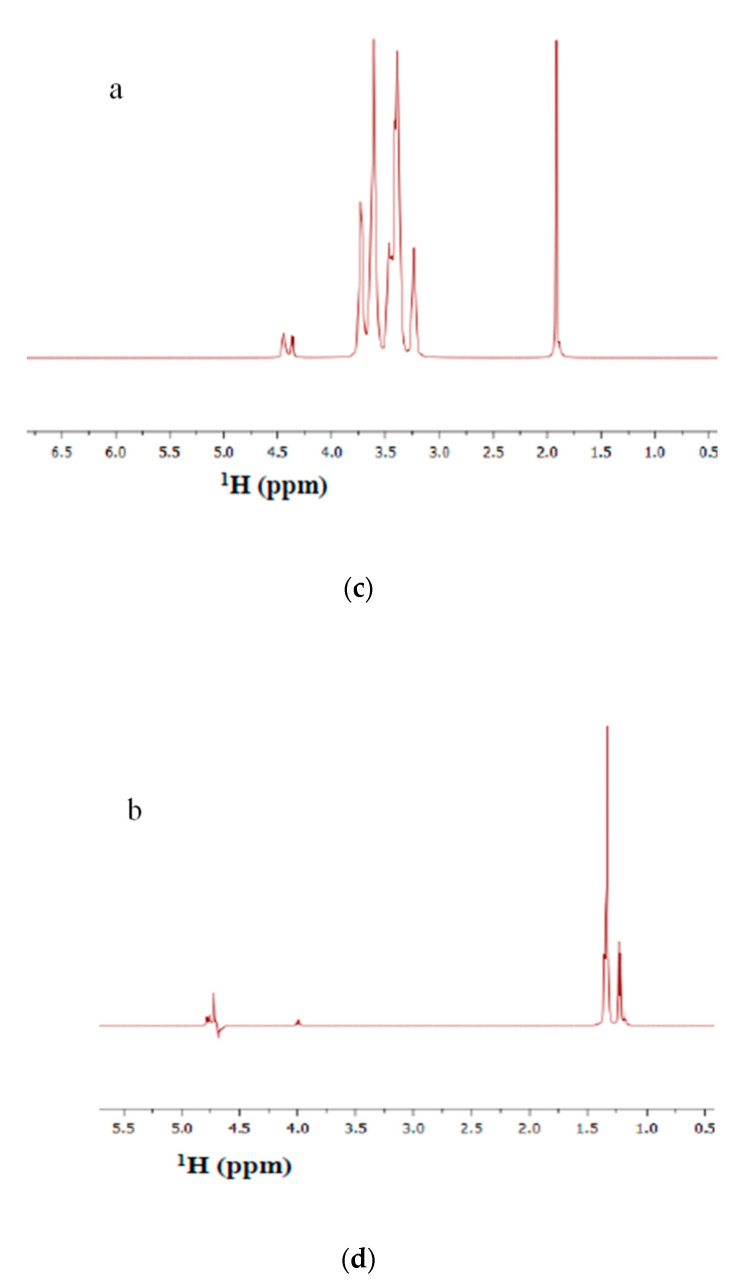
(**a**) NMR spectrum of the sample of T15F1 of HA-PLA DAC^®^ sterilized and degraded as in Table 1 as reported in Materials and Methods. (**b**) DOSY spectrum of the sample T15F1. The DOSY indicates the presence of two diffusive fronts due to chemical species “**a**” and “**b**”. Front “**a**” represents the residual HA polymer with a MW slightly decreased with respect to the result in Figure 3, showing D = 1.30 × 10^−10^ m^2^ s^−1^ corresponding to a MW of 37 kDa. Front “**b**” represents the fragments of PLA released by hydrolysis of the conjugated bridge. The region of the vertical profile circled in red shows the presence of the fragments with a higher diffusivity (3.9–2.50 × 10^−9^ m^2^ s^−1^). The region circled in blue is attributed to the diffusive front of the residual deuterium hydrogen oxide HDO at 4.76 ppm. For D_2_O residual protons, the value of D found was 3.79 × 10^−9^ m^2^ s^−1^. (**c**) Slice of front “**a**” of the DOSY spectrum. (**d**) Slice of front “**b**” of the DOSY spectrum.

**Figure 7:**
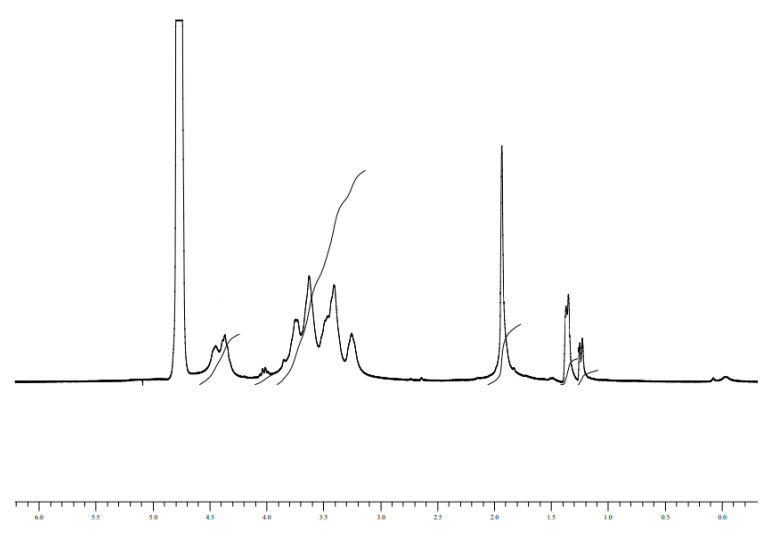
NMR spectrum of sample T7F1 in D2O.

The stability studies have been performed by NMR and with DOSY NMR upon the time on the samples reported in Table 1. The NMR and DOSY of sample T15F1 are reported in Figure 6a–d and the NMR spectrum of T7F1 in D_2_O are displayed in Figure 7.

In the spectrum in Figure 6a, the decrease of the resonance at 5.2 ppm and the rising of the resonances characteristic of the degradation of PLA into dimers and trimers of lactic acid are evident (Monomer: (CH3) δ 1.23; (CH) δ 4.03; Dimer: (CH3) δ 1.27–1.38; (CH) δ 4.19–4.92; Trimer: (CH3) δ 1.27–1.40–1.44; (CH) δ 4.20–4.98–5.10) [16]. Moreover, DOSY experiments showed evidently that different diffusive fronts due to different hydrodynamic radius are present (a) HA resonances without the resonances characteristic of PLA, (b) the resonances due to PLA moiety and the resonances due to oligomers of lactic acid.

In Figure 6b the left projection circled in red displays species with higher diffusivity than the (a) trace and, then with lower MW than the HA residual. These are also visible in the left projections of the DOSY spectrum, circled in red.

In Figure 6c,d the slices (a) and (b) of the DOSY in Figure 6b are reported and correspond to the spectral profiles of HA and PLA fragments. Furthermore, the diffusion coefficient of the HA polymer for this last study time appears close to that of the HA standard at 50 kDa.

The spectrum of the sample T7F1 in Figure 7 indicates that the ester bond is nearly completely hydrolyzed, and the PLA is completely transformed in lactic monomer, dimer, and trimer, for this study time.

### 3.3. Study by Gel Permeation Chromatography

After the analysis of the reference standards necessary to obtain the reference values, different batches of the sterile DAC^®^ has been used at a fixed concentration of 1.0 mg/mL. The retention values (R.t.) found are reported in Table 2. The study required us to perform a separate experiment to describe correctly the hydrolysis behavior of the sterile DAC^®^ recovered from the titanium disk surface (D samples) or, alternatively, from bulk exposed to biofluids (simplified as PBS buffer) (F samples). Thus, two series of samples have been studied D and F all in triplicate as listed in Table 1.

An important result is that the two D and F series show a similar behavior thus indicating that the proximity to the titanium disk surface it is not able to induce differences in the chemistry of the degradation.

The obtained results indicated that when the ester bond undergoes hydrolysis, the degraded samples lose progressively the amphiphilic character thus decreasing the micellar aggregation and decreasing the retention time significantly.

The chromatographic analysis results can be summarized as follows: the sterilized HA-PLA DAC^®^ shows an apparent MW lower than that measured of the non-sterilized product; the retention time trend at the different study times suggests that hydrolysis occurs at the ester bond between HA and PLA.

In fact, the retention values (Table 2) have a clear decreasing trend upon the time of exposure to hydrolysis thus confirming what was reported in NMR study’s section for HA-PLA DAC^®^ with and without sterilization.

In summary, upon time the hydrolysis of the ester bond between HA and PLA increases thus decreasing the marked amphiphilic character of the copolymer and, then, decreasing the tendency to form micellar aggregation.

At the time T15 a single polymeric species is present identified as HA-Na with an apparent MW ≥ 50 kDa.

**Figure 8:**
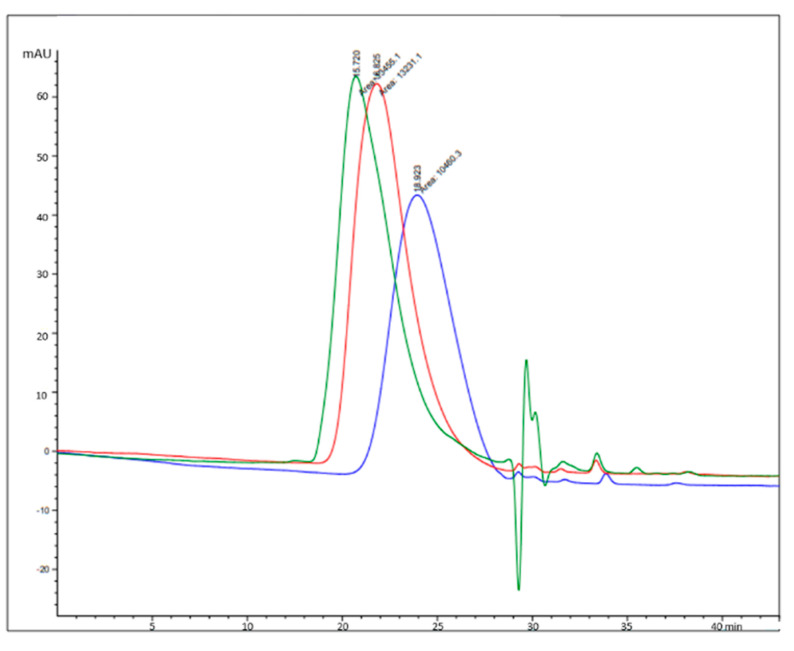
Overlay of the chromatograms of sample HA-Na 13 kDa (blue), Ha-Na 50 kDa (red), and T15F1 (green). In the horizontal axis the retention times in minutes are reported.

In Figure 8, the chromatograms of: HA-Na 13 kDa (blue), Ha-Na 50 kDa (red), and T15F1 (green) are reported, displaying a comparable apparent MW for the T15 sample with HA standard at 50 kDa. All the experiments were performed in triplicate as reported in Materials and Methods and the results showed an excellent reproducibility.

### 3.4. Considerations about Diffusion Data

Taking into consideration the limits of precision in the determination of the absolute value of diffusion coefficients by DOSY due to the available strength of the gradient of the 700 MHz instrument (6G), these results are an interesting example of the utility of this technique for the study of polymer fragmentation. In particular, the diffusion measure of the HA standards proved to discriminate properly the different MWs, considering that a good comparison can be performed for species possessing the same molecular characteristics. In fact, this is a recommendation formulated [29] for a good comparison of the molecular weights, where the use of similar structures eliminates systematic errors related to the use of standards with different chemical features.

In the diffusion measurements of DAC^®^ in D_2_O, the unique front shown in Figure 3 has a D = 1.23 × 10^−9^ m^2^ s^−1^. The values of the diffusive fronts in Figure 6b, are as follows: front a) D = 1.30 × 10^−10^ m^2^ s^−1^ and front b) (fragment distribution) from 3.0 to 2.54 × 10^−9^ m^2^ s^−1^.

In the case of HA certified standard fragments the diffusion coefficients were: 13 kDa D = 2.22 × 10^−10^ m^2^ s^−1^; 50 kDa D = 1.11 × 10^−10^ m^2^ s^−1^; 208 kDa D = 5.18 × 10^−11^ m^2^ s^−1^.

Moreover, a detailed study of the possible interferences between these types of polymers was carried out. In fact, the possibility of entangling should be further investigated. These experiments consist of the DOSY measure of the mix of two standard fragments with different MWs. This was made by observing DOSY in the diffusion coefficient of the same standards using either a mix of the 13 kDa and the 208 kDa samples and the mix of the 50 kDa and 208 kDa. These experiments are reported in Figures S2 and S3.

In the first case (13 kDa and 208 kDa) the diffusion coefficients of the diffusive fronts were D = 2.29 × 10^−10^ m^2^ s^−1^ for the 13 kDa fragments and 1.20 × 10^−10^ m^2^ s^−1^ for the 50 kDa instead of the same fragments alone which were 2.22 × 10^−10^ m^2^ s^−1^ and 1.11 × 10^−10^ m^2^ s^−1^ respectively.

The other experiment was performed on a mix of the 13 kDa and the 208 kDa fragments. Results were for the 13 kDa front D = 4.07 × 10^−10^ m^2^ s^−1^ and for the 208 kDa one D = 4.09 × 10^−11^ m^2^ s^−1^. Additionally, in this case, a difference is clearly visible between the diffusion D of the fragment alone reported above and the same in the mixture.

In fact, in the case of fragments alone for 13 kDa D = 2.22 × 10^−10^ m^2^ s^−1^ and for 208 kDa D = 5.18 × 10^−11^ m^2^ s^−1^. The differences are not very large but are indicative that the presence of other macromolecules in the solution can affect the absolute value of the diffusion coefficient. This can be also due either to entangling between macromolecules or to an effect of the change in solution viscosity.

The diffusion of water in these systems (plot of Figure 3) was monitored. The D value of the trace of the deuterium hydrogen oxide (HDO) resonance was 1.86 × 10^−9^ m^2^ s^−1^.

On the other hand, in Figure 6b the diffusion front of a resonance at 4.76 ppm due to HDO shows a value of 3.79 × 10^−9^ m^2^ s^−1^. This is the value experimentally determined in the presence of HA-PLA macromolecules and the fragments upon fragmentation. Moreover, the D value of the resonance of HDO molecule in the mix of fragments reported above shows a variable value of D of HDO, in fact in the mix of 13 kDa and 208 kDa D was measured in 4.45 × 10^−9^ m^2^ s^−1^. On the basis of the literature diffusion of net water as a trace of the protonated water molecule, in practice HDO (1% of H_2_O in D_2_O) a value D = 1.12 × 10^−9^ m^2^ s^−1^ is reported also with NMR [37,38,39] and with other physical techniques [40,41]. The variability of the diffusion of the HDO molecule is evidently strongly affected by the size and the dispersity of the macromolecules in the solution. It is also important to consider that HA contains a large number of hydroxyl groups able to alter the property of protons of HDO by the prototropic exchange.

In addition, the preliminary experiments included also the use of a High MW hyaluronic acid purchased as reported in Materials and Methods. The MW was with a value about MW ≥ 500 kDa.

The results are not suitable for publication due to the bad quality of the very noisy spectra but it is interesting that the diffusive profiles on the left of the DOSY spectra indicate the presence of three broad peaks with D = 1.69 × 10^−11^ m^2^ s^−1^, 2.51 × 10^−11^ m^2^ s^−1^ and 4.68 × 10^−11^ m^2^ s^−1^. In the same experiment, the lactic acid molecule (100 Da) gave a value of 4.3 × 10^−9^ m^2^ s^−1^.

## 4. Conclusions

From the results of chromatographic analysis, HPLC-GPC-UV and NMR spectroscopy included DOSY NMR of sterilized HA-PLA DAC^®^ it is possible to conclude that the sterilization process obtained with ionizing radiation induces a reduction of MW of HA-PLA DAC^®^ as already reported by several authors.

Moreover, HA-PLA DAC^®^ sterilized in physiological conditions (PBS), as in the proprietary Novagenit protocols for checking stability, shows the expected behavior obtaining a complete hydrolysis after about 15 days and the degradation into monomeric, dimeric, and trimeric species within the sensitivity of the techniques used.

The copolymer HA-PLA DAC^®^ sterilized after 15 days in the PBS stability test shows an apparent MW of almost 50 kDa in both the techniques applied. The sterilized HA-PLA DAC^®^ shows an amphiphilic character with micellar aggregation properties in the polar media as indicated by the results in agreement for all the applied techniques.

Finally, in our case, the diffusion studies by DOSY NMR spectroscopy confirm to be a very useful tool in investigating the behavior of chemically conjugated and bioconjugated compounds in pharmaceutical industry research.

## Figures and Tables

**Table 1:** The 24 samples of HA-PLA (Novagenit DAC^®^) recovered for HPLC and NMR study both from gel on disk and in buffer solution as indicated (see text).

Time Days	Name	Quantity (mg)	Origin	Name	Quantity (mg)	Origin
0	T0D1	227.2	Gel on disk	-	-	Buffer solution
0	T0D2	169.9		-	-	
0	T0D3	163.2		-	-	
1	T1D1	192.3		T1F1	709.3	
1	T1D2	212.3		T1F2	654.9	
1	T1D3	210.2		T1F3	668.7	
3	T3D1	126.2		T3F1	727.8	
3	T3D2	134		T3F2	754.6	
3	T3D3	126.8		T3F3	733	
7	T7D1	7		T7F1	839.4	
7	T7D2	8		T7F2	818.4	
7	T7D3	7.2		T7F3	829.1	
15	T15D1	-		T15F1	893.1	
15	T15D2	-		T15F2	830.3	
15	T15D3	-		T15F3	848.3	

**Table 2:** Chromatographic retention values of samples of Table 1 examined as reported in Materials and Methods.

Name	Quantity (mg)	H_2_O (mL)	Concentration (mg/mL)	Retention Time (min)
T0-D1	4.3	4.300	1.00	17.07
T0-D2	6	6.000	1.00	17.39
T0-D3	7.1	7.100	1.00	17.24
T1-D1	2.5	2.500	1.00	17.17
T1-D2	3.3	3.300	1.00	16.68
T1-D3	2.3	2.300	1.00	16.24
T1-F1	8.2	1.171	7.00	16.08
T1-F2	8	1.143	7.00	16.40
T1-F3	7.9	1.129	7.00	16.01
T3-D1	9	9.000	1.00	16.32
T3-D2	4.5	4.500	1.00	15.9
T3-D3	9.6	9.600	1.00	16.01
T3-F1	8	1.143	7.00	15.71
T3-F2	9	1.286	7.00	15.77
T3-F3	9.3	1.329	7.00	15.72
T7-D1	1.3	1.300	1.00	15.87
T7-D2	2.5	2.500	1.00	15.81
T7-D3	3	3.000	1.00	15.84
T7-F1	8.7	1.243	7.00	15.68
T7-F2	8.6	1.229	7.00	15.66
T7-F3	8.4	1.200	7.00	15.65
T15-F1	7.7	1.100	7.00	15.72
T15-F2	7.6	1.086	7.00	15.69
T15-F3	7.8	1.114	7.00	15.70

## Supplementary Materials

The following are available online at https://www.mdpi.com/2218-273X/10/11/1478/s1, Figure S1: Behaviour of HA-PLA conjugate DAC^®^ after sterilization in different solvent mixture indicated, Figure S2: DOSY experiments conducted as reported in Materials and Methods on the mixture of two fragments of HA with different MWs, Figure S3: DOSY experiments conducted as reported in Materials and Methods on the mixture of two fragments of HA with different MWs.
