# Supplementary material for: Stability Evaluation and Degradation Studies of DAC® Hyaluronic-Polylactide Based Hydrogel by DOSY NMR Spectroscopy
Source: Biomolecules. 2020 Oct 24;10(11):1478. doi: 10.3390/biom10111478 (PMC7690892; doi:10.3390/biom10111478)
Supplement: Supplementary file 1 [file biomolecules-10-01478-s001.pdf]

Supplementary Materials

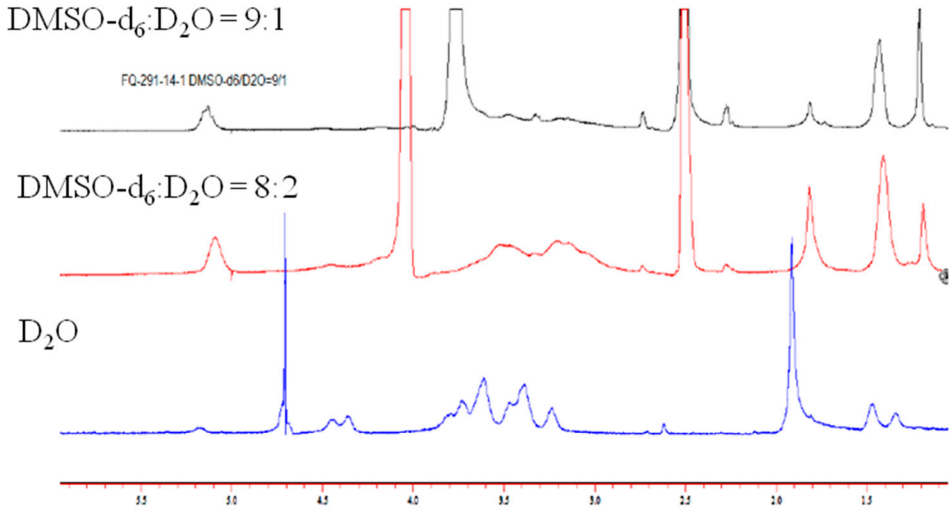

Figure S1. Behaviour of HA-PLA coniugate DAC® after sterilization in different solvent mixture indicated.

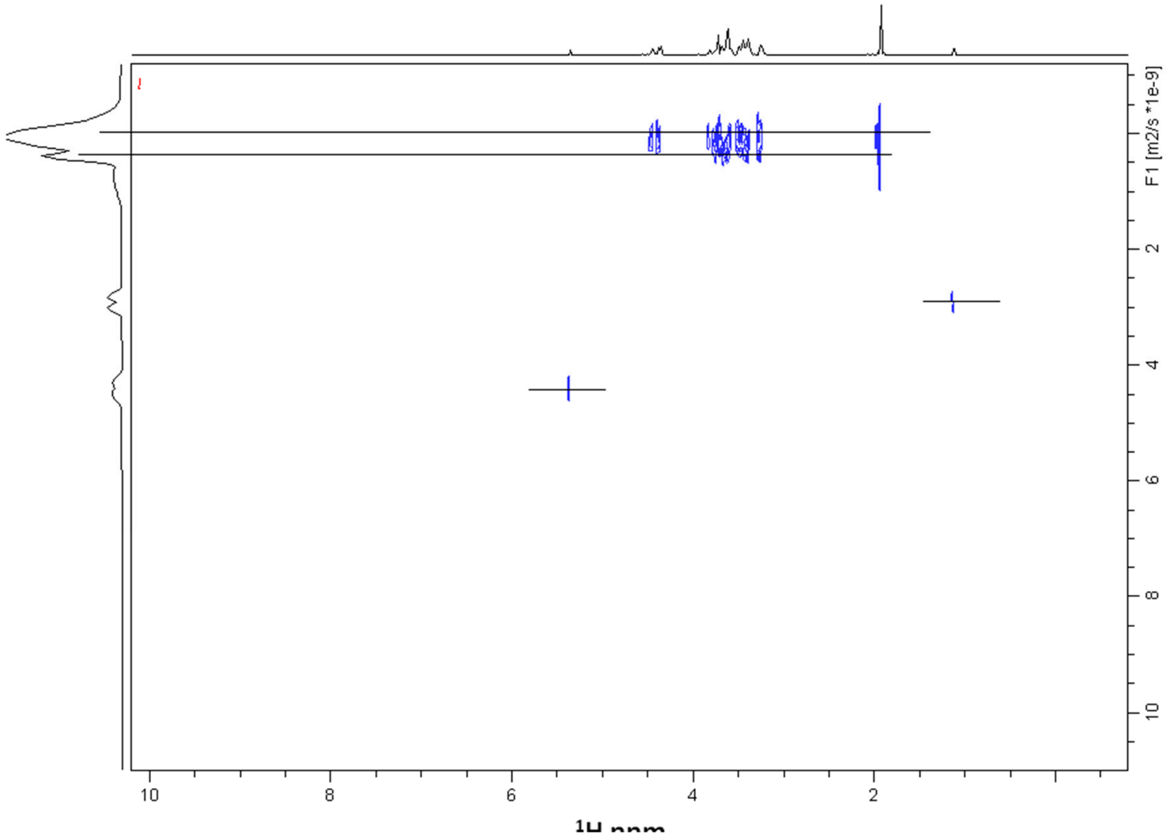

Figure S2. DOSY experiments conducted as reported in Materials and Methods on the mixture of two fragments of HA with different MWs. Mix of HA 13 kDa and HA 208 kDa.

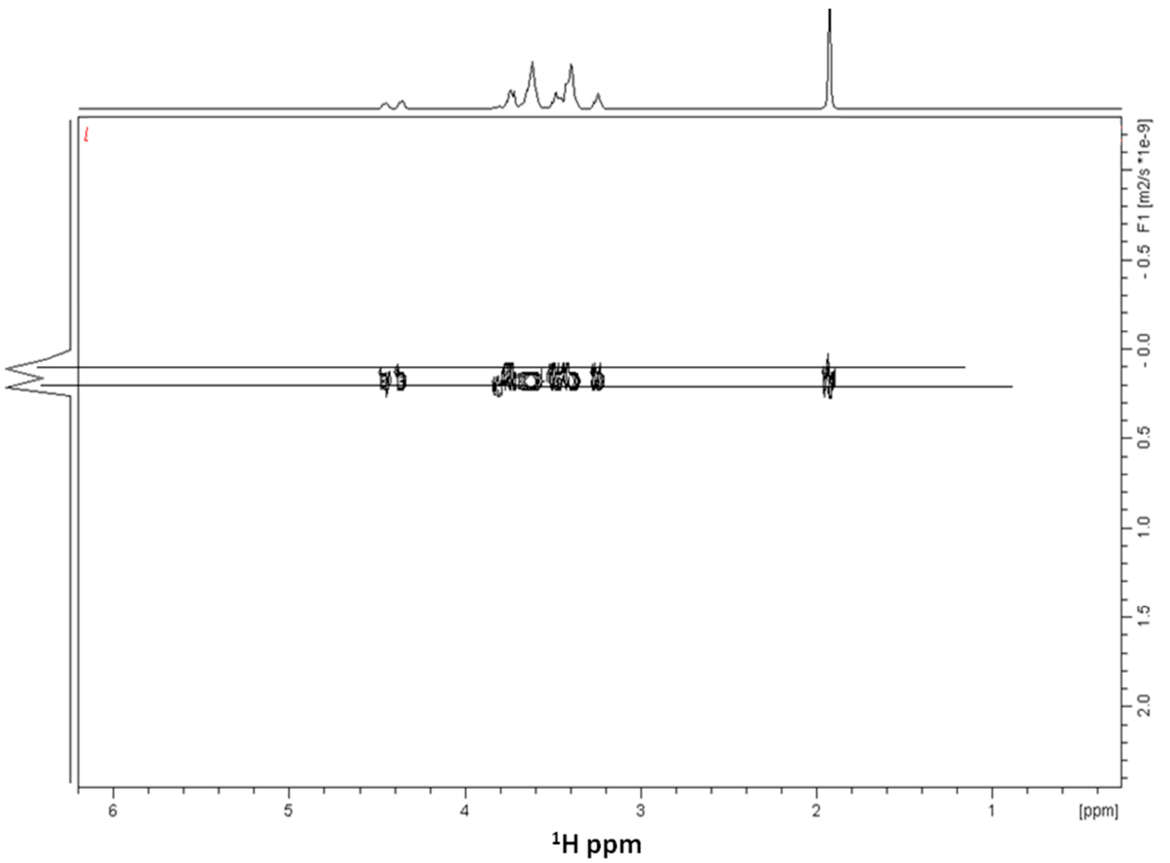

Figure S3. DOSY experiments conducted as reported in Materials and Methods on the mixture of two fragments of HA with different MWs. Mix of HA 13 kDa and HA 50 kDa.
